# Implementing the collaborative chronic care model for mental health: a nonrandomized clinical trial

**DOI:** 10.1186/s43058-026-00995-y

**Published:** 2026-06-22

**Authors:** Christopher J. Miller, Jennifer L. Sullivan, Kelly Stolzmann, Rebecca A. Raciborski, Madisen Brown, Hannah M. Bailey, Samantha L. Connolly, Alexandra L. Morena, Maura Dougherty, Alexandria Henderson, Andrea Rehmert, Lauren M. Sippel, Bo Kim

**Affiliations:** 1 Center for Health Optimization and Implementation Research (CHOIR), VA Boston Healthcare System (152 M), 150 S. Huntington Ave., Boston, MA 02130 USA; 2 Department of Psychiatry, Harvard Medical School, Boston, MA USA; 3 Transformative Health Systems Research to Improve Veteran Equity and Independence Center of Innovation (THRIVE COIN), VA Providence Healthcare System, Providence, RI USA; 4 Department of Health Services, Policy and Practice, Brown University School of Public Health, Providence, RI USA; 5 Behavioral Health Quality Enhancement Research Initiative (QUERI), Central Arkansas Veterans Healthcare System, North Little Rock, AR USA; 6 VA Office of Mental Health, Washington, DC USA; 7 VA New England Program Evaluation Center (NEPEC), West Haven, CT USA

**Keywords:** Collaborative chronic care models, Outpatient mental health, Implementation facilitation, Centralized technical assistance

## Abstract

**Background:**

Collaborative chronic care models (CCMs) are an evidence-based way to structure care in outpatient mental health settings. This project investigated whether implementation facilitation (IF) can enhance CCM-consistent outpatient mental health care and reduce mental health hospitalizations compared with centralized technical assistance (CTA).

**Methods:**

This was a stepped wedge hybrid 2 implementation-effectiveness trial in outpatient mental health clinics at eight US Department of Veterans Affairs (VA) medical centers (four waves, two sites each) conducted from 2022 to 2024. Staff participants included clinicians involved in delivering team-based outpatient mental health care. The patient sample consisted of US military veterans treated by 42 participating mental health teams. IF was used for 8–9 months to increase alignment with CCM principles at each participating site. All the sites (including eight comparison sites that were nonrandomized but matched on key variables) had access to CTA. The coprimary outcomes were (a) alignment with CCM-based care evaluated using the Role Clarity and Team Primacy subscales of the Team Development Measure [TDM], and (b) mental health hospitalizations extracted from the electronic health record during the year following initiation of IF.

**Results:**

The sample included *n* = 20,835 patients at participating sites and *n* = 60,221 patients at comparison sites. The pre-implementation TDM was completed by 134 staff members, and post-implementation TDM was completed by 109 staff members. Role Clarity and Team Primacy subscales of the TDM demonstrated small, nonstatistically significant improvement after completing IF (*p* = 0.33[*t*-value: 0.97] and 0.14 [*t*-value: 1.48], respectively). Multivariate generalized linear mixed models indicated participating sites had similar mental health hospitalization rates following IF initiation compared to sites receiving CTA alone.

**Conclusion:**

This trial revealed that IF to enhance CCM-consistent clinical practices in outpatient mental health teams was associated with small improvements in team functioning that did not achieve statistical significance. Mental health hospitalization rates did not differ for sites using IF versus CTA. TDM-based assessments of team functioning at baseline were similar to post-implementation results from a prior trial, suggesting that CTA to support team-based care may have increased team functioning in these settings prior to trial initiation.

**Trial registration:**

NCT05997836. Retrospectively registered 18 August 2023: https://clinicaltrials.gov/study/NCT05997836?term=NCT05997836%26rank=1. Ethical approval: This evaluation was conducted as a quality improvement activity for VA and was deemed by the R&D Committee at the VA Boston Healthcare System not to be research; therefore, it was not subject to IRB review.

**Supplementary information:**

The online version contains supplementary material available at https://doi.org/10.1186/s43058-026-00995-y.

Contributions to the literature

- Comparisons between implementation strategies are needed to help healthcare system leaders decide how to allocate scarce resources.
- This project revealed that implementation facilitation, a relatively resource-intensive implementation strategy, did not outperform centralized technical assistance in helping outpatient mental health teams align their care practices with the collaborative chronic care model (CCM).
- The results highlight the value of system-wide implementation efforts to enhance the functioning of outpatient mental health teams.

## Background

Collaborative chronic care models (CCMs) represent an evidence-based approach to structuring care for chronic conditions including mental health conditions [1]. CCMs incorporate some or all of six elements to promote more anticipatory, continuous, and well-coordinated care. These elements include work role redesign to ensure that staff roles support care coordination; self-management support for patients; clinical decision support for providers; clinical information systems to support panel-based care and data tracking; linkages to community resources outside the health system; and consistent leadership support [2].

Despite the evidence base for CCMs for management of mental health conditions, implementing and sustaining them in busy clinical practices remains challenging. Much of the literature has focused on sustainability of collaborative mental health care practices in primary care settings [3–5]. When it comes to CCM implementation in mental health settings specifically, a previous implementation trial in the US Department of Veterans Affairs (VA) found improvements in team functioning and care quality following the use of implementation facilitation (IF) [3] to support the uptake of CCM-based care practices in nine outpatient mental health teams [6]. Follow-up analyses revealed no evidence that gains had been sustained in the four years following implementation [7–9].

Since that trial was conducted, the VA Office of Mental Health (OMH) has provided centralized technical assistance (CTA) to support more widespread adoption of CCM-consistent care practices in mental health care teams at VA medical centers across the country. CTA activities have included dissemination of a workbook and implementation checklist to guide CCM uptake; availability of brief consultation; national training for outpatient mental health leaders and staff conducted in 2023; establishment of a SharePoint site containing guiding documents and data tools; three national communities of practice; and regional communities of practice to foster interfacility planning and idea generation. As a result, many VA sites entered the current study period with access to a relatively mature, system-wide infrastructure to support CCM implementation. In this context, the present study was designed to evaluate the incremental value of IF beyond these existing supports, rather than testing IF in the absence of system-level infrastructure. This distinction is important, as the effectiveness of more intensive implementation strategies may depend on the level of baseline support already available within a healthcare system.

This hybrid implementation-effectiveness trial had two goals. First, given limited sustainability of CCM-based care in our prior trial [7–9], we adapted IF to increase the sustainability of a CCM for outpatient mental health teams. These adaptations were based on quantitative and qualitative data from that trial [10], and involved having facilitators working at the level of the outpatient mental health service (rather than just one outpatient mental health team per clinic). The adapted form of IF also involved having the site’s internal facilitator take a more prominent role in the implementation process (rather than having the implementation process driven primarily by the external facilitator; see Methods below for details) [10].

Second, we planned to compare this adapted version of IF to the CTA that is now widely available to VA sites looking to implement collaborative outpatient general mental health teams with care practices rooted in the CCM (i.e., Behavioral Health Interdisciplinary Program or BHIP teams; details below). Our specific goal was to evaluate whether IF provides benefit beyond already available system-wide implementation support infrastructure. We hypothesized that IF would outperform CTA: in this manuscript, we present implementation and clinical results from data collected one year after the initiation of IF at eight VA medical centers.

## Methods

### Overview

This evaluation was conducted as a quality improvement activity for VA and was deemed by the R&D Committee at the VA Boston Healthcare System not to be research; therefore, it was not subject to IRB review. It was structured as a hybrid implementation-effectiveness trial [11] and registered as a clinical trial in ClinicalTrials.gov (NCT05997836). As described below, the study was designed as a stepped wedge, but neither co-primary analysis relied on the stratified random assignment of participating sites to study waves. Thus, this study meets the criteria for a nonrandomized quasi-experimental study. The project followed the EQUATOR recommendations for reporting implementation research [12]; see Supplements 1 and 2 for details. The CCM constituted the evidence-based practice—in this case, an evidence-based way to deliver care within the participating outpatient mental health clinics—and IF and CTA constituted the implementation strategies (details below).

### Clinical setting

This project was conducted in the context of VA general outpatient mental health clinics. Starting in 2013, OMH^1^ began moving toward a team-based model for care delivery in these settings [13]. This involved establishing interdisciplinary care (i.e., BHIP) teams at VA medical centers nationally. Each BHIP team is intended to include a minimum of eight licensed and non-licensed clinical staff (e.g., psychiatrists, psychologists, social workers, nurses, peer support, and care management staff), along with administrative support, with a panel of about 1,000 US military veteran patients per team. In 2015, OMH formally adopted the CCM as the guiding clinical model for BHIP teams nationally.

### Site selection

We recruited eight sites to participate, providing 80% statistical power to detect 10-point changes over time in percent agreement on relevant Team Development Measure subscale scores, as well as differential change in hospitalization rates of 0.25% (described respectively under Implementation Outcomes and Clinical Effectiveness outcomes below). As with our previous trial [6], we recruited sites in close partnership with OMH, with the intention of selecting sites that were (a) geographically diverse, (b) willing to commit resources to BHIP implementation, and (c) in need of assistance per their own self-assessment. Participating sites completed and signed a Program Letter of Agreement that outlined project expectations (Supplement 3). For mental health hospitalization analyses, we selected eight VA comparison sites that were closely matched to our eight participating sites on key facility-level variables (Supplement 4) [14], but did not undergo IF.

### Implementation strategies

#### Implementation facilitation (IF)

Participating sites underwent 8–9 months of IF [3], an interactive strategy intended to support the uptake of complex evidence-based clinical practices into routine care settings [15]. In this case, IF featured an external facilitator (study-funded; authors C.J.M. and B.K.) who provided expertise on CCM implementation and quality improvement techniques. The facility internal facilitator, in contrast, was facility-funded and named in the Program Letter of Agreement as dedicating an estimated 4 hours/week to leading the implementation process. Typically, internal facilitators were mental health leaders with process improvement experience at their medical center.

IF was built around a set of core activities [16] and included a thorough pre-implementation assessment; introductory meetings with site staff; 8–9 months of weekly virtual meetings between the internal and external facilitators; and larger meetings (typically 1–4 times per month) including both facilitators and additional site staff involved in the implementation process (e.g., mental health leaders, administrators, BHIP team leads, data analysts, and support staff). This process was guided by the *BHIP-CCM Enhancement Guide*, a workbook maintained by OMH [17] that includes guidance for developing more CCM-consistent care practices for BHIP teams.

As described in more detail elsewhere [10], our implementation facilitation approach (alongside the *BHIP-CCM Enhancement Guide*) was revised prior to trial initiation to enhance the sustainability of the changes made during the IF period at each participating site. These adaptations were made based on quantitative and qualitative data showing sub-optimal sustainability of CCM-based clinical practices from that trial [10], and included: working at the level of the outpatient mental health service (rather than just one BHIP team); shifting the locus of control for the implementation process more firmly to the site itself (rather than having the external facilitator be seen as driving the implementation process); and shortening the IF period to 8–9 months (rather than six months of active facilitation and six months of stepdown). Additionally, our previous implementation trial featured a 1.5-day, in-person site visit at each site: this site visit transitioned to a set of virtual introductory meetings at each site. These adaptations were informed by the Iterative Decision-making for Evaluation of Adaptations (IDEA) [18] and were documented via the Framework for Reporting Adaptations and Modifications to Evidence-based Implementation Strategies (FRAME-IS) [19]. Based on consensus panel recommendations regarding core facilitation activities [16], these adaptations were considered to be fidelity-consistent.

The internal facilitator time commitment to the project was estimated to be four hours per week per site. External facilitator time tracking [20] indicated that each external facilitator spent a mean ± SD of 64 ± 12.7 hours (range 40–84 hours, for an average of about two hours per week) per site; this time tracking also confirmed that the external facilitators engaged in core implementation facilitation activities for the pre-implementation and implementation phases [16]. When accounting for staff salaries, this suggested that IF cost the health system about $20,000 ± $4,000 per site.

#### Centralized technical assistance (CTA)

All VA medical centers had access to CTA for the duration of the trial. As part of CTA, OMH maintains a SharePoint site (accessible VA-wide) that includes the *BHIP-CCM Enhancement Guide* alongside other implementation resources to support CCM-consistent care practices among BHIP teams. In 2022, OMH disseminated a memo package and BHIP-CCM implementation checklist requiring VA medical centers to implement BHIP-CCM and document their BHIP-related care practices on a phased, facility-directed schedule between fiscal years 2022 and 2027. OMH staff also led a weeklong BHIP-CCM national training in 2023 involving outpatient mental health leaders and clinicians from every VA regional network. Beginning in 2023, OMH developed three monthly national communities of practice and a national consultation program by which medical centers could request brief implementation support. OMH has also supported the setup of regional communities of practice to foster interfacility planning, idea generation, and the sharing of promising practices. As part of CTA, OMH also maintains a suite of dashboards to allow BHIP teams to track caseloads, identify veterans in need of follow-up, and inform care coordination activities. Thus, CTA represents a robust comparison implementation strategy that includes multiple components, although it was not as intensive on a per-site basis as IF.

Participation in CTA components was not systematically tracked at the site level for all elements (e.g., communities of practice attendance, dashboard use). However, CTA resources, including national training events, communities of practice, consultation opportunities, and data dashboards, were broadly available across VA sites, and project team communications and operational records indicate that both participating and comparison sites engaged with these resources to varying degrees during the study period. Given the system-wide availability of CTA, some degree of overlap in exposure between participating and comparison sites was expected.

### Stepped wedge design

We employed a cluster stepped wedge design to enhance internal validity while also ensuring that all participating sites received IF [21], consistent with the needs of OMH. Participating sites were assigned to one of four different start times (heretofore labeled Waves 1 through 4) about three months apart (start dates: May 2023 through January 2024; last site completed October 2024). Assignment to waves was done via stratified randomization, employing a balancing algorithm [14] to minimize imbalance between waves (based primarily on the same facility-level variables that were used to select comparison sites; see Supplement 4). For each participating site, baseline corresponded to the start of the IF period; outcomes for the comparison sites were assessed at the same time as the participating sites to avoid temporal confounding. Because implementation and clinical outcomes (described below) do not rely on the stratified randomization of sites to waves, we conceptualize the study as a quasiexperimental nonrandomized design despite the inclusion of the stepped wedge. All sites had access to CTA throughout the trial, provided by OMH as described above.

### Participant population and sample

The patient population of interest mirrored that of our prior work [6, 22]. Patients were considered to be treated by a participating BHIP team at the start of IF (and therefore included in the study sample) if they had at least two outpatient mental health visits to that outpatient clinic in the prior year, including at least one visit in the previous three months. Patients diagnosed with dementia were excluded. We used this same approach to identify patients treated by the matched comparison sites that had ongoing access to CTA.

Each IF site had between three and twelve BHIP teams considered in scope for this project (identified at the outset of IF for each site; 42 teams in total across all eight participating sites). The clinician population of interest included all frontline clinicians assigned to a participating BHIP team.

### Implementation outcomes

The primary implementation outcome was the Team Development Measure [23], which includes four subscales related to delivery of collaborative, team-based care. Cohesiveness assesses how well team members get along with one another; Communication assesses the quality and frequency of within-team communication; Role Clarity assesses the extent to which team members know and value the roles of different team members; and Team Primacy assesses team members’ willingness to prioritize team goals over individual goals. All items are answered on a four-point Likert scale ranging from “Strongly Disagree” to “Strongly Agree;” subscale scores were calculated as percentages of “Agree” and “Strongly Agree” responses. From the perspective of Proctor and colleagues [24], we considered the TDM to represent a measure of CCM adoption, as our prior work [6] indicated that TDM scores correlate very highly (*r* above 0.90) with more exhaustive measures of CCM-based care delivery.^2^ Furthermore, that work indicated that the Cohesiveness and Communication subscales may be subject to a ceiling effect due to being relatively high at baseline [6]. Thus, our a priori hypothesis was that IF would be associated with significant increases from pre- to post-IF in the Role Clarity and Team Primacy subscales. TDM analyses were within-site only; based on project scope we did not administer the TDM at comparison sites.

### Clinical effectiveness outcomes

The primary clinical outcome was the number of mental health hospitalizations experienced by patients treated by participating BHIP teams compared to those experienced by patients treated at comparison sites. Hospitalization data were derived from the VA Corporate Data Warehouse [25], which includes data on hospitalizations in VA settings and the Integrated Veteran Care Consolidated Data Set [26] (IVC CDS), which contains data on veteran hospitalizations paid for by the VA that occurred in non-VA settings. Hospitalization rates were calculated quarterly, starting one year before baseline at each site and ending one year after initiation of IF (i.e., for eight consecutive quarters, coded as yes/no for each veteran within each quarter). Our a priori hypothesis was that we would detect statistically significant change in mental health hospitalization rates after 8–9 months of IF compared to the change in those rates at the comparison sites undergoing CTA alone.

### Statistical analysis

Demographic and clinical characteristics were compared for patients treated at the facilities that underwent IF to the comparison medical centers. This was done using c^2^ tests for categorical variables and *F* tests from one-way ANOVA for continuous variables. TDM results (primary implementation outcome) were calculated from baseline to post-implementation (i.e., comparing baseline to 8–9 months later) within participating sites only and analyzed using unpaired 2-tailed *t* tests (as some clinicians completed one TDM and others completed both administrations, and we did not collect identifiers for staff participants that would have allowed calculation of paired sample *t* tests).

Mental health hospitalization results (our primary clinical effectiveness outcome) were analyzed using generalized linear mixed models with site-level random intercepts and patients nested in sites and cluster-robust standard errors. A linear spline model with a knot at baseline and an interaction term with site type (participating site vs. control site, 0/1) was included to determine whether participating sites’ change from baseline to post-implementation differed from the analogous change for comparison sites. Three facility-level covariates (facility complexity [27], clinical mental health providers per 1,000 unique mental health patients, and total administrative support per mental health provider) were also included in adjusted hospitalization models. Patient analyses were considered intention-to-treat in that patient variables were drawn from administrative data (such that there were no patient dropouts). Analyses were conducted using SAS EG version 8.3 (SAS Institute Inc.) and Stata version 18 (StataCorp LLC, College Station, TX).

## Results

### Study flow and participant characteristics

**Fig. 1:**
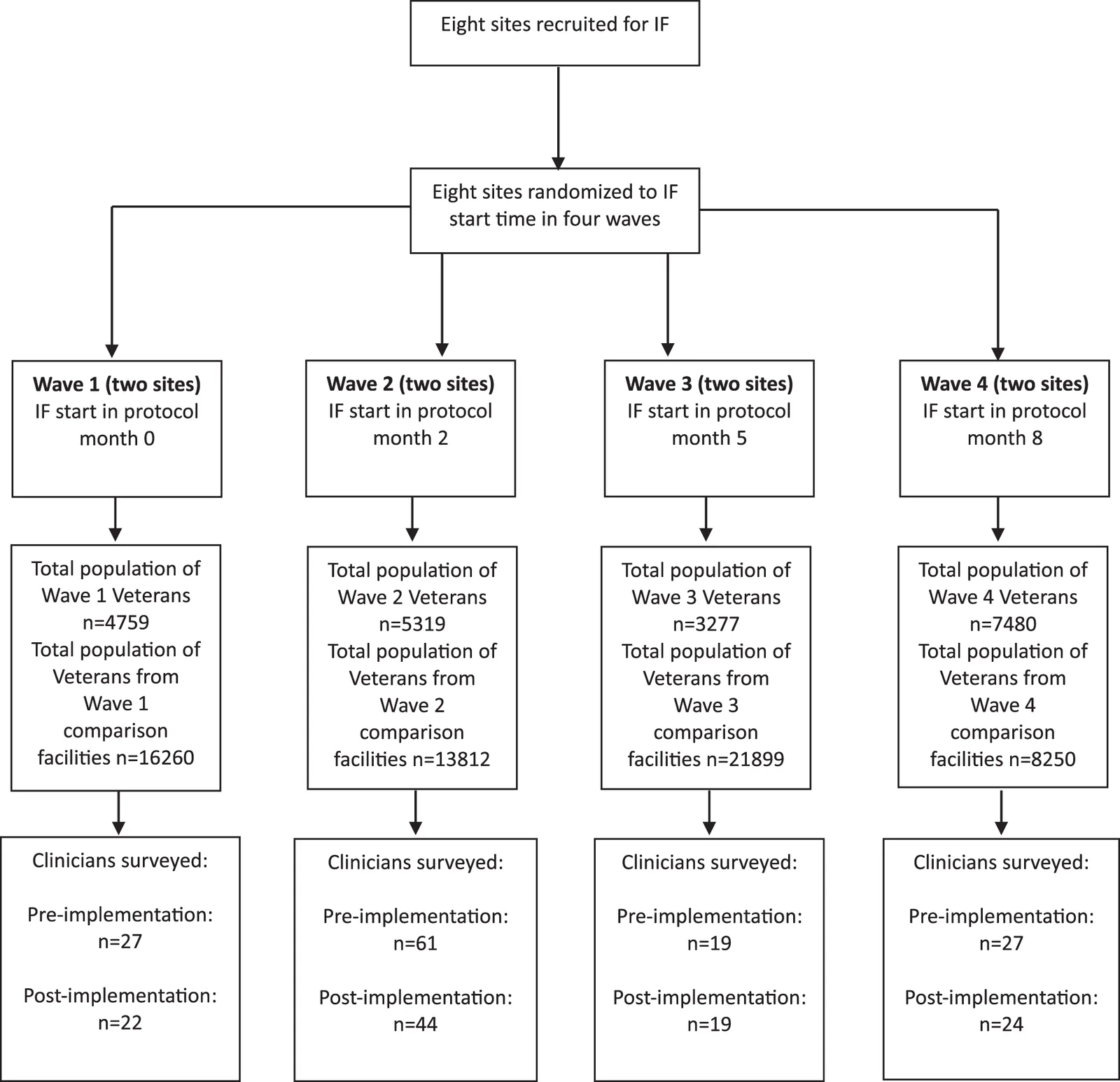
CONSORT diagram for facilities, veterans, and clinicians

Overall study flow is represented in Fig. 1. We identified 369 BHIP team members at participating sites at baseline, and 431 at post-implementation. Of these, 134 clinicians completed the TDM at baseline (37% response rate), whereas 109 completed it post-implementation (26% response rate). Site-level response rates ranged from 12% to 67% at baseline, and from 16% to 51% post-implementation. The baseline sample included 25% psychologists, 13% psychiatrists, 30% social workers, 20% nurses, and 12% other professionals. The postimplementation sample included 18% psychologists, 18% psychiatrists, 26% social workers, 16% nurses, and 22% other professionals.

The patient sample across the eight participating sites (*n* = 20,835) was 54 ± 16 years old (mean ± SD), and 16% female (Table 1). Patients at participating sites were more likely to be White or Hispanic than at comparison sites (*n* = 60,221). Given the large number of patients, differences in other demographic or clinical variables between the two groups were generally statistically significant but small in magnitude.

**Table 1:** Baseline veteran participant characteristics

Variable	Population treated within CCM-enhanced teams at participating sites (*n* = 20835)	Population treated at matched comparison sites (*n* = 60221)^a^	Comparison χ^2^ or *F* test	*p* value	Effect size^b^
Demographic data					
Age, mean (SD)	54.35 (16.03)	53.07 (15.47)	104.634	<0.0001	0.00
Female gender, number (%)	3,239 (15.55)	12,407 (20.6)	254.084	<0.0001	−0.06
Race/ethnicity, number (%)			2422.61	<0.0001	0.18
White	14,588 (79.11)	34,591 (62.69)			
Black	2,539 (13.77)	17,899 (32.44)			
Other	1,313 (7.12)	2,684 (4.86)			
Hispanic (%)	2,525 (13.23)	5,368 (9.43)	222.08	<0.0001	0.05
Minority (%)	6,136 (31.44)	25,357 (43.77)	919.584	<0.0001	−0.11
Married, number (%)	9,921 (48.44)	30,027 (50.52)	26.4	<0.0001	−0.02
Employed (full- or part-time; self-employed), number (%)	7,010 (38.93)	17,486 (31.39)	348.965	<0.0001	0.07
Rural residence, number (%)	6,747 (32.38)	21,087 (35.02)	47.763	<0.0001	−0.02
VA disability ≥ 50%, number (%)	15,815 (88.22)	48,514 (90.41)	70.694	<0.0001	−0.03
Clinical data, number (%)					
Depression or anxiety disorder	13,606 (65.3)	37,962 (63.04)	34.335	<0.0001	0.02
Serious mental illness (bipolar spectrum or schizophrenia)	2,694 (12.93)	8,296 (13.78)	9.447	0.00211	−0.01
Posttraumatic stress disorder	11,643 (55.88)	32,669 (54.25)	16.665	<0.0001	0.01
Substance use disorder	4,138 (19.86)	11,025 (18.31)	24.557	<0.0001	0.02
No. active medical diagnoses, mean (SD)^c^	0.81 (1.07)	0.76 (1.03)	34.061	<0.0001	0.00
No. active mental health diagnoses, mean (SD)^d^	1.86 (1.03)	1.78 (0.99)	114.699	<0.0001	0.00
Mental health hospitalization in prior year (within VA) (%)	775 (3.72)	2,228 (3.7)	0.017	0.89521	0.00
Mental health hospitalization in prior year (outside VA) (%)	229 (1.1)	863 (1.43)	12.988	<0.001	−0.01
Medical-surgical hospitalization in prior year (within VA) (%)	1,131 (5.43)	3,269 (5.43)	0	0.9999	0.00

Patients treated by between 3 and 12 BHIP teams were included for participating sites, and patients treated by all BHIP teams were included for comparison sites

Effect sizes for continuous variables are presented as η^2^, and categorical variables as Cramer’s V

Among eight potential medical diagnoses: diabetes, obesity, hyperlipidemia, cardiac dysrhythmia, liver disorder, kidney disorder, thyroid disorder, and traumatic brain injury

Among seven potential mental health diagnoses: bipolar disorder, schizophrenia spectrum disorders, alcohol and/or drug use disorders, anxiety disorders, posttraumatic stress disorder, attention deficit hyperactivity disorder, and major depressive disorder

### Implementation outcomes

TDM subscales are reported in Table 2. As initially hypothesized, Cohesiveness and Communication scores were relatively high at baseline (above 80% Agree or Strongly Agree responses) and did not improve significantly at post-IF. Percent Agree or Strongly Agree responses for Role Clarity and Team Primacy improved by a mean of 3.7 (95% CI: −3.9 to 11.3) and 6.3 (−2.1 to 14.7) percentage points from baseline to post-IF, respectively: these findings were small in magnitude (Cohen’s d ≤ 0.20) and were not statistically significant at the *p* < 0.05 level. To determine whether these results might differ based on response rates, we conducted a sensitivity analysis for the five sites with at least a 25% response rate across both TDM administrations. Results from this analysis were substantively similar to results for the full sample.

**Table 2:** Team Development Measure (TDM) subscale scores at participating sites (baseline and post-IF)

TDM subscale	Baseline (Pre-IF; *n* = 134), % (95% CI)	Post-IF (*n* = 109), % (95% CI)	Change, percentage points (95% CI)	*p* value	Cohen’s d (95% CI)
Cohesiveness	85.7 (81.5–90.0)	86.6 (81.3–92.0)	0.90 (−5.8, 7.6)	0.79	0.04 (−0.23–0.30)
Communication	89.3 (85.4–93.2)	90.9 (86.6–95.2)	1.6 (−4.1, 7.3)	0.58	0.07 (−0.19–0.34)
Role Clarity	69.1 (64.1–74.1)	72.8 (67.1–78.6)	3.7 (−3.9, 11.3)	0.33	0.13 (−0.13–0.39)
Team Primacy	65.8 (60.0–71.7)	72.1 (66.1–78.2)	6.3 (−2.1, 14.7)	0.14	0.20 (−0.06–0.46)

### Clinical effectiveness outcomes

**Fig. 2:**
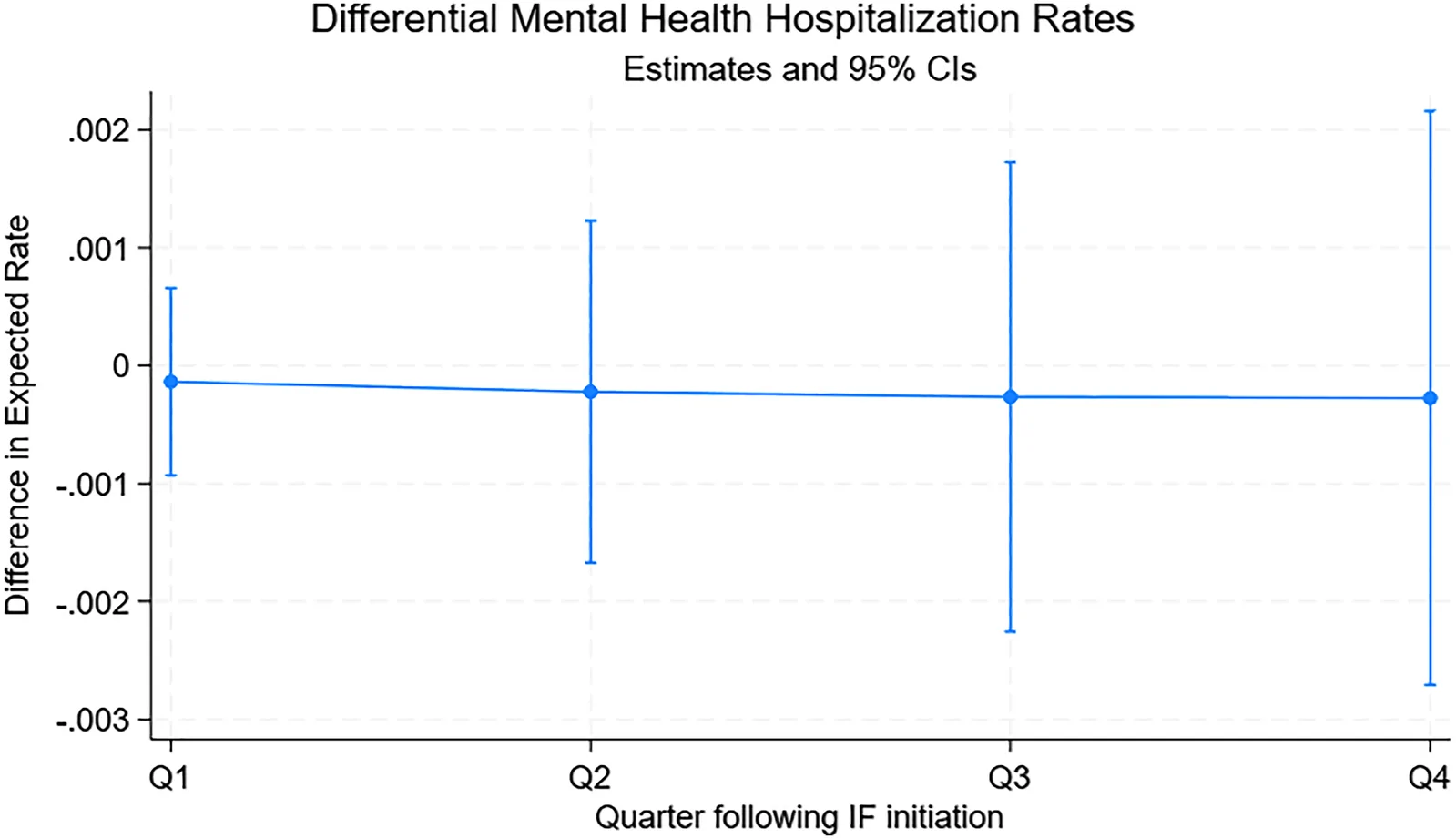
Differences in predicted probability of mental health hospitalization for sites assigned to implementation facilitation versus centralized technical assistance

Implementation facilitation was not associated with statistically significant change in mental health hospitalizations over the entirety of the one-year period following baseline compared to the matched comparison sites using CTA alone (Fig. 2). Specifically, differences in predicted probability of hospitalization between IF and CTA sites fell in the range of 0.0001 to 0.0003 per quarter favoring IF sites in multivariate models (joint chi-square = 5.84; *p* = 0.12). Based on our previous findings—in which reductions in mental health hospitalizations emerged about six months into the implementation period [4]—we repeated these analyses for the period 7–12 months after IF initiation (i.e. the latter six months of the one-year period following baseline). Similar to the results for the full year, however, these findings slightly favored IF sites but were not statistically significant (*p* = 0.14). Full multivariate logit model results, including hospitalization odds and robust standard errors, are provided in Supplementary File 5.

## Discussion

Collaborative Chronic Care Models (CCMs) are an evidence-based approach for structuring outpatient mental health care. Implementing and sustaining CCM-based care in real-world clinical settings, however, is challenging. In this manuscript, we present post-implementation outcomes from a hybrid implementation-effectiveness trial that used two implementation strategies for enhancing uptake of CCM-consistent care: implementation facilitation (IF) and centralized technical assistance (CTA). Based on our stepped wedge design, eight participating sites had access to CTA prior to initiating IF, and matched comparison sites had access to CTA resources for the duration of the trial. This study represents an ambitious expansion of prior work [6], focused on multiple frontline BHIP teams at each of eight medical centers (rather than one team per facility), and featuring sustainability-focused adaptations to IF [10].

The primary implementation outcome was a change in two subscales (Role Clarity and Team Primacy) of the Team Development Measure (TDM) over time at IF sites. In prior work, TDM scores correlated strongly with more resource-intensive assessments of CCM-based care. In the current study, the results indicated small improvements in these TDM domains that did not reach our prespecified 95% threshold for statistical significance. This suggests that IF was only minimally effective at achieving stated goals of improving the extent to which BHIP team members understood and appreciated one another’s clinical roles and increasing emphasis on shared team goals.

We note, however, that baseline levels of TDM Role Clarity and Team Primacy were 15–20 percentage points higher than in our prior work [6]. For a relevant comparison, baseline TDM scores for the current study were nearly identical to post-IF TDM scores in that previous trial. We therefore surmise that CTA—including ongoing national and regional BHIP-CCM Community of Practice calls; a SharePoint site with BHIP-related resources; and a four-day, national in-person training conducted in 2023—has been associated with substantial improvements in team functioning over the years prior to initiation of IF at participating sites. Consistent with this, while not formally part of our analysis plan, we note that OMH addressed BHIP-related consultation requests from at least five of the eight comparison sites during the trial. While these developments are welcome, they may have contributed to a saturation effect for this trial, suggesting that CTA may have essentially raised the “floor” for participating sites in a way that made it more difficult for IF to have noticeable further impact. Other large-scale implementation trials have similarly shown limited incremental benefit of resource-intensive facilitation approaches [28]. For example, in an implementation trial of a CCM in community practices in Michigan and Colorado, offering more facilitation resources did not result in better performance than lower-resource support models for sites where CCM-based practices had already taken root [29]. Such findings suggest diminishing marginal returns for higher-intensity support in systems where baseline readiness or prior exposure to implementation infrastructure is high.

To further contextualize these findings, we consider for whom and under what conditions IF may be most effective while interpreting results in terms of effect magnitude and precision. The observed improvements in Role Clarity and Team Primacy were small in absolute terms and accompanied by wide confidence intervals, suggesting that, while meaningful improvements cannot be ruled out, the most likely effects are modest. In this context, the absence of statistical significance should not be interpreted as evidence of no effect, but rather as reflecting limited precision in estimating relatively small changes.

Drawing on the Integrated Promoting Action on Research Implementation in Health Services (i-PARIHS) framework [30], which emphasizes the interplay between context and facilitation in shaping implementation outcomes, the effectiveness of IF relative to CTA is likely contingent on baseline contextual conditions and the extent to which facilitation addresses locally salient barriers. In this study, available CTA, including national training, communities of practice, consultation resources, and data infrastructure, likely elevated baseline levels of CCM-consistent practices and team functioning. In such contexts, even small effect sizes may reflect diminishing returns on additional facilitation effort, particularly for outcomes approaching ceiling levels. Conversely, in lower-readiness or lower-infrastructure settings, similarly sized effects may be more meaningful, as modest improvements in team functioning may represent important progress and reflect facilitation addressing barriers not fully mitigated by existing supports. Taken together, these findings underscore the importance of tailoring implementation strategies to local context and suggest that more intensive facilitation resources may be most efficiently targeted to sites with greater contextual need.

These findings highlight an important opportunity for future research to more explicitly define and measure implementation strategy saturation. In particular, there is a need to identify indicators of when systems have reached a level of implementation maturity at which additional support yields diminishing returns, as well as to better understand which specific system-level actions (e.g., training, communities of practice, consultation infrastructure) contribute most to achieving such conditions. Advancing this line of inquiry could inform more efficient allocation of implementation resources by helping determine when higher-intensity strategies are warranted and when lower-intensity, system-level supports may be sufficient.

Our study’s primary clinical effectiveness outcome was probability of mental health hospitalization among patients treated by BHIP teams from participating IF sites compared to patients treated by BHIP teams at matched comparison sites using CTA alone. To be clear, if a veteran patient experiences acute psychiatric symptoms, then inpatient hospitalization may be the most appropriate setting in which to receive care.—But, ideally, CCM-consistent care will increase the extent to which patients can achieve wellness with less intensive outpatient treatments. The estimated effect from a mixed-effects model of hospitalization did not reveal statistically significant differences in predicted probability of hospitalization aggregated over the four quarters after implementation between the IF and comparison sites. Additional analyses focused specifically on the period 7–12 months following initiation of IF revealed similar results, with the predicted probability of mental health hospitalization at IF sites being only slightly lower than predicted probability at sites using CTA.

The lack of statistical significance in the trend needs additional contextualization. First, we note that the estimated trend is the average effect for each quarter; it does not capture the cumulative effect of the change. It is possible that, while quarter-over-quarter changes are small, the cumulative effect over multiple quarters is more substantial. Second, we included only a limited set of the most relevant site-level characteristics; unmeasured facility-level factors were included in the random intercept. While the estimated effect of IF should still be unbiased, it is estimated with more noise because of this decision. Additionally, our study used a small number of clusters (16 total) with many observations per cluster (~2,600 on average at participating sites and ~7,500 at comparison sites). Simulation analyses have revealed that hypothesis tests may result in a rejection rate that is above the nominal level in such situations [31].

In aggregate, the findings above are lower in magnitude than those of our prior work [6]. Our original trial involved working with one BHIP team per medical center, treating an aggregated patient population of 5,596 veteran patients across nine sites. At the time of the original trial, the BHIP model of care was still very nascent, and the comparison group consisted primarily of clinicians who had not been delivering team-based care at all. In the current study, IF focused on multiple BHIP teams at each medical center (a total of 42 BHIP teams treating over 20,000 veteran patients across eight sites). The decision to broaden the scope of the project in this way was made in part to enhance sustainability of CCM-based care at these sites, as making changes across multiple BHIP teams at each medical center would hopefully minimize the impact of any one given clinician retiring or otherwise leaving the clinic [10]. But it is possible that this may have diluted the impact of IF at each participating site.

We also note that, unlike our previous trial, comparison sites for the current study included BHIP teams with access to CTA resources to support collaborative work. Thus, comparison sites may have had more developed BHIP teams than did those from our previous trial, which may partly explain why IF did not outperform CTA in this trial. The modest gains observed in this trial may reflect the notion of multi-level contextual factors moderating implementation effectiveness as reflected in established implementation determinants frameworks (e.g., i-PARIHS, Consolidated Framework for Implementation Research) [30, 32], which emphasize the role of contextual factors (e.g., existing infrastructure) in shaping whether and how implementation strategies yield desired outcomes. Future quantitative analyses will examine the cost implications of these findings, as well as the sustainability of these findings at one-year follow-up, which align with the continued and growing emphasis in the field on understanding which strategies are most cost-effective and appropriate given site readiness and existing support structures [33, 34]. Upcoming qualitative analyses from this trial will provide additional information regarding the reasons that IF may have worked better at some sites than others.

### Limitations

We note several limitations of these findings. First, this was a cluster-randomized stepped wedge trial, for which the primary clinical effectiveness outcome (mental health hospitalizations) relied on a nonrandomized set of matched comparison sites. This design could lead to a biased estimate of the effect in the hospitalization models, especially given the small number of clusters, if the cohort effect is confounded with site-specific characteristics. To address this, we controlled for site characteristics in adjusted models and entered site as a random effect. Even so, it is possible that IF may have been associated with changes in other, less distal clinical measures (e.g. depression severity) that we did not assess. Second, model fit in our adjusted mental health hospitalization models, as assessed by positive predictive power, was generally poor. This is a result of how unpredictable rare events like hospitalization are at the individual level. In addition to the other factors that may have influenced the precision of the estimated effect of IF, this statistical noise also likely reduced our ability to detect quarter-over-quarter change at the 95% statistical significance level. Third, we did not administer the TDM to staff at matched comparison sites and instead analyzed pre- and post-implementation administrations of the TDM at participating sites. However, the stepped wedge design (featuring staggered start times for sites) made it unlikely that secular trends alone would explain our results. Fourth, the response rate for the TDM was relatively low, with about 30% of eligible BHIP team members completing it at each administration, and with significant variability in response rates across sites. However, sensitivity analyses (focused on sites with relatively higher response rates) revealed findings that were substantively similar to those for the full sample. Fifth, beyond its response rate, we acknowledge that the TDM itself was not initially developed as an implementation outcome measure (although it correlated very highly with more exhaustive assessments of CCM adoption in our prior work [6]). This study could have been strengthened had we been able to include additional measures of CCM implementation. Sixth, we did not systematically collect detailed quantitative data on site-level engagement with CTA components (e.g., communities of practice participation, dashboard use), which limited our ability to characterize variation in CTA exposure across sites or formally assess the extent of overlap between participating and comparison sites and its potential influence on study findings. Seventh, this was a non-research study conducted in one integrated health system (the US Department of Veterans Affairs). We urge caution in generalizing these findings to other health systems. And finally, we note that this trial was registered in clinicaltrials.gov retrospectively (about three months after trial initiation). However, no data were analyzed and no study design elements were changed between the start of the trial and the successful registration.

### Conclusions and future directions

Collaborative Chronic Care Models (CCMs) are designed to support more anticipatory, continuous, and evidence-based care that is delivered in a thoughtfully coordinated manner. This stepped wedge trial compared two different implementation strategies, implementation facilitation (IF) and centralized technical assistance (CTA), to help VA outpatient mental health teams deliver more CCM-consistent mental health care. Results indicated that IF was not associated with statistically significant improvements when compared to CTA. However, comparisons to our prior trial suggest that this may in part reflect a saturation effect (i.e. the lack of statistical differentiation may imply that IF had a limited impact above and beyond the gains that had already been made under CTA, which does not imply that IF is necessarily ineffective), and clarifying how to identify and respond to implementation strategy saturation represents an important direction for future implementation research. Ongoing qualitative analyses will attempt to identify key factors influencing uptake of CCM-based care at participating sites; economic analyses will evaluate the cost implications of these different approaches to implementing CCM-based care; and sustainability analyses will assess the extent to which measures of team functioning or mental health hospitalizations change in the year following completion of IF at participating sites.

## Electronic supplementary material

Below is the link to the electronic supplementary material.

Supplementary Material 1

Supplementary Material 2

Supplementary Material 3

Supplementary Material 4

Supplementary Material 5

Supplementary Material 6

## Data Availability

The dataset is not publicly available due to confidentiality policies.

## Footnotes

1 BHIP implementation was initially launched by the VA Office of Mental Health and Suicide Prevention (OMHSP). In 2023, OMHSP split into the VA Office of Mental Health (OMH) and the VA Office of Suicide Prevention (OSP). At that point, OMH maintained oversight of the BHIP Program. Throughout the manuscript was use the OMH acronym to enhance readability.

2 The Team Development Measure may alternatively be considered a measure of service outcomes rather than implementation outcomes. Given that it correlated very highly with CCM adoption in our previous trial, however, we conceptualize it here as our primary implementation outcome.
