# Supplementary material for: Implementing the collaborative chronic care model for mental health: a nonrandomized clinical trial
Source: Implement Sci Commun. 2026 Jun 22;7:180. doi: 10.1186/s43058-026-00995-y (PMC13548575; doi:10.1186/s43058-026-00995-y)
Supplement: Supplementary file 3 [file 43058_2026_995_MOESM4_ESM.docx]

**Supplemental File 4: Variables Used for Site Balancing and Matching**

| **Variable Name** | **Variable used for assigning sites to waves (balancing)?** | **Variable used for matching participating sites to comparison sites?** | **Participating Sites (n=8)** | **Comparison Sites (n=8)** | **p value (t-test or chi-sq) for Participating vs. Comparison Sites** |
| --- | --- | --- | --- | --- | --- |
| Mean encounters with outpatient general mental health staff in Fiscal Year 2022 | Yes | Yes | 88632 | 89447 | 0.9797 |
| Percent of mental health encounters conducted by telephone | Yes | Yes | 23.4 | 21.4 | 0.6019 |
| Percent of mental health encounters conducted via video telehealth | Yes | Yes | 30.3 | 33.5 | 0.5636 |
| Psychological Safety aggregate score from VA All Employee Survey (on 1-5 scale, with 1 being worst and 5 being best) | Yes | Yes | 3.87 | 3.89 | 0.6589 |
| VA Facility Complexity (percent) | Yes | Yes |  |  | 0.8874 |
| 1a High Complexity |  |  | 50 | 38 |  |
| 1b High Complexity |  |  | 0 | 13 |  |
| 1c High Complexity |  |  | 25 | 25 |  |
| 2 Medium Complexity |  |  | 13 | 13 |  |
| 3 Low Complexity |  |  | 13 | 13 |  |
| Percent of facilities serving primarily rural Veterans | Yes | Yes | 38 | 13 | 0.2482 |
| Region (%)* | Yes | Yes |  |  | 0.7847 |
| Midwest |  |  | 13 | 0 |  |
| Northeast |  |  | 25 | 13 |  |
| Southeast |  |  | 25 | 38 |  |
| Southwest |  |  | 25 | 38 |  |
| West |  |  | 13 | 13 |  |
| Planned Start Date for BHIP-CCM implementation (mean date, ranging from 2022 to 2026) | No | Yes | 1/20/2023 | 4/25/2023 | 0.2887 |
