# Supplementary material for: Implementing the collaborative chronic care model for mental health: a nonrandomized clinical trial
Source: Implement Sci Commun. 2026 Jun 22;7:180. doi: 10.1186/s43058-026-00995-y (PMC13548575; doi:10.1186/s43058-026-00995-y)
Supplement: Supplementary file 4 [file 43058_2026_995_MOESM5_ESM.docx]

**Supplementary File 5:**

**Multivariable Logit and Standard Error Estimates for Mental Health Hospitalization**

| **Variable** | **Logit (se):**  **entire facilitation year** | **p-value: entire facilitation year** | **Logit (se):**  **7-12 months after initiation of IF** | **p-value: 7-12 months after initiation of IF** |
| --- | --- | --- | --- | --- |
| Time (knot at T0) | -0.112 (0.006) | <0.0001 | -0.363 (0.015) | <0.0001 |
| Facilitation Site | -0.169 (0.177) | 0.340 | -0.187 (0.185) | 0.312 |
| Facilitation Site*Time (knot at T0) | -0.031 (0.028) | 0.264 | -0.153 (0.094) | 0.104 |
| Female | -0.223 (0.044) | <0.0001 | -0.192 (0.045) | <0.0001 |
| Race (ref=Other) |  |  |  |  |
| White | -0.033 (0.065) | 0.614 | -0.082 (0.093) | 0.379 |
| Black | 0.192 (0.057) | 0.0008 | 0.130 (0.090) | 0.149 |
| Hispanic | -0.038 (0.033) | 0.245 | 0.051 (0.042) | 0.228 |
| Married | -0.364 (0.039) | <0.0001 | -0.308 (0.042) | <0.0001 |
| Employed | -0.472 (0.052) | <0.0001 | -0.473 (0.052) | <0.0001 |
| Rural residence | -0.234 (0.069) | 0.0007 | -0.214 (0.066) | 0.001 |
| VA Disability >= 50% | -0.263 (0.069) | 0.0001 | -0.296 (0.068) | <0.0001 |
| Depression or Anxiety | 0.008 (0.050) | 0.875 | 0.050 (0.051) | 0.329 |
| Serious mental illness (bipolar spectrum or schizophrenia) | 1.124 (0.046) | <0.0001 | 1.135 (0.044) | <0.0001 |
| Posttraumatic stress disorder | -0.209 (0.060) | 0.0006 | -0.207 (0.065) | 0.001 |
| Substance use disorder | 1.338 (0.074) | <0.0001 | 1.399 (0.082) | <0.0001 |
| Medical-surgical hospitalization in prior year (within VA) | 0.759 (0.101) | <0.0001 | 0.731 (0.104) | <0.0001 |
| Age | -0.023 (0.002) | <0.0001 | -0.023 (0.002) | <0.0001 |
| No. active mental health diagnoses | 0.427 (0.036) | <0.0001 | 0.456 (0.037) | <0.0001 |
| No. active medical diagnoses | 0.244 (0.061) | <0.0001 | 0.284 (0.066) | <0.0001 |
| No. active medical diagnoses (sq) | -0.029 (0.013) | 0.034 | -0.035 (0.014) | 0.014 |
| Facility Level: Complexity (ref=3) |  |  |  |  |
| 1 | 0.046 (0.319) | 0.886 | 0.106 (0.350) | 0.762 |
| 2 | 0.936 (0.352) | 0.008 | 1.055 (0.389) | 0.007 |
| Facility-level: MH Providers | -0.020 (0.037) | 0.598 | -0.017 (0.038) | 0.656 |
| Facility Level: Admin Support | -0.509 (0.965) | 0.597 | -0.343 (1.064) | 0.747 |
| Intercept | -4.239 (0.875) | 0.001 | -4.467 (0.972) | 0.001 |

Note: logit refers to mental health hospitalization rates for the IF period versus comparison sites accessing CTA (negative values indicate lower hospitalization rates at IF sites).
