# Supplementary material for: Implementing the collaborative chronic care model for mental health: a nonrandomized clinical trial
Source: Implement Sci Commun. 2026 Jun 22;7:180. doi: 10.1186/s43058-026-00995-y (PMC13548575; doi:10.1186/s43058-026-00995-y)
Supplement: Supplementary file 5 [file 43058_2026_995_MOESM6_ESM.docx]

**Supplementary File 6: List of Abbreviations**

| BH QUERI | Behavioral Health Quality Enhancement Research Initiative |
| --- | --- |
| BHIP | Behavioral Health Interdisciplinary Program |
| CHOIR | Center for Health Optimization and Implementation Research |
| CCM | Collaborative chronic care model |
| CTA | centralized technical assistance |
| FRAME-IS | Framework for Reporting Adaptations and Modifications to Evidence-based Implementation Strategies |
| IDEA | Iterative Decision-making for Evaluation of Adaptations |
| IF | implementation facilitation |
| NEPEC | VA New England Program Evaluation Center |
| OMH | VA Office of Mental Health |
| OMHSP | VA Office of Mental Health and Suicide Prevention |
| TDM | Team Development Measure |
| VA | US Department of Veterans Affairs |
