# Supplementary material for: Implementing the collaborative chronic care model for mental health: a nonrandomized clinical trial
Source: Implement Sci Commun. 2026 Jun 22;7:180. doi: 10.1186/s43058-026-00995-y (PMC13548575; doi:10.1186/s43058-026-00995-y)
Supplement: Supplementary file 6 [file 43058_2026_995_MOESM3_ESM.doc]

**Program Letter of Agreement**

***Behavioral Health Interdisciplinary Program Collaborative Chronic Care Model (BHIP-CCM)***

***Enhancement Project 2.0***

**BHIP-CCM Enhancement Project 2.0 Lead:**

Christopher Miller, PhD; Center for Health Optimization & Implementation Research (CHOIR); VA Boston Healthcare System ([christopher.miller8@va.gov](mailto:christopher.miller8@va.gov); phone: XXX-XXX-XXXX)

**Project Summary:**

This project is a quality improvement (non-research) project funded by the VA Behavioral Health Quality Enhancement Research Initiative (**BH QUERI**) and supported by the VA Office of Mental Health and Suicide Prevention (**OMHSP**).

In 2013, OMHSP set the target of establishing one Behavioral Health Interdisciplinary Program team (**BHIP**) in every VA Medical Center. In 2015, OMHSP adopted the Collaborative Chronic Care Model (**CCM**) as the guiding clinical model for BHIP teams. OMHSP guidance now requires all VA Medical Centers and Community Based Outpatient Clinics (CBOCs) to adopt the BHIP-CCM model of care—that is, BHIP teams built around CCM-consistent care practices—throughout all of their general mental health clinics over the next five years. This project’s goal is to help VA Medical Centers adopt this model of care during a focused 8-month implementation period.

**BH QUERI/OMHSP Contribution:**

The implementation team, led by Dr. Miller, has extensive experience in evidence-based implementation to establish new programs, using external facilitators (Dr. Miller and Dr. Bo Kim) working with an Internal Facilitator (Medical Center staff member) and other Medical Center staff. This specific BHIP-CCM implementation project will support team cohesion and organize BHIP care processes according to the CCM principles, guided by the recently updated *BHIP-CCM Enhancement Guide*.

Implementation will consist of 8 months of active work with the Medical Center, including:

- Pre-implementation assessment of local BHIP strengths and opportunities for improvement
- Virtual kickoff meeting to review CCM elements and potential applications to BHIP teams within the Medical Center
- Weekly & ad hoc process improvement consultation for the Internal Facilitator and representatives from each BHIP team
- Regular feedback to mental health service and Medical Center leadership

The project is expected to reach eight Medical Centers in FY2023-2024, with the start date for each Medical Center sequenced randomly to begin over the next 8-10 months. Program impact data will be compiled by the implementation team from several sources, including BHIP monitoring data collected by OMHSP. There are also optional staff survey and interview components to this project, which are voluntary and confidential for BHIP clinicians and other staff.

**Medical Center Contribution:**

To enhance post-implementation sustainability of BHIP-CCM, the Medical Center agrees to provide the following support during the 8-month implementation period (please check boxes):

- Identification of BHIP teams’ members and clinics (expectation is that implementation will occur across multiple BHIP teams simultaneously)
- Allocation of one hour per week for at least one representative from each local BHIP team (generally the BHIP Team Lead), along with any additional local BHIP program managers or Champions (ideally 4-12 representatives in total, constituting a **BHIP Implementation Team**); this time allocation (spanning 6+ months) will be used for BHIP-CCM process improvement work
- Blocking of BHIP teams’ schedules for BHIP team meetings (at least one hour per meeting, to discuss shared clinical cases and process improvement decisions stemming from the BHIP Implementation Team mentioned immediately above) at least 3 times per month
- Medical Center or VISN staff member, ideally with mental health experience and process improvement experience, to serve as Internal Facilitator for at least 6 months at 8-10% effort. The intention is for the Internal Facilitator to be the primary point of contact for the External Facilitator at the site.

Internal Facilitator name: ______________________________________

Planned Facilitation Period (start / end dates): _____________________________

- A BHIP Program Manager and/or Mental Health Service Lead with oversight responsibility for the BHIP team(s), who will support the enhancement effort internally as a **BHIP Champion** on an as-needed basis (e.g. monthly to demonstrate ongoing support for the initiative)

BHIP Champion name: ______________________________________

- Agreement that this overall change effort will be led by the Internal Facilitator and BHIP Implementation Team, with consultation from the External Facilitator; from previous experience and research, we realize that sustainability is most likely if the effort is driven primarily by the Internal Facilitator and local BHIP Implementation Team, rather than the “external consultants”
- Agreement that the BHIP Implementation Team will be responsible for disseminating process redesign decisions to their local BHIP teams to enact the process redesign described above and providing active feedback on which further refinements would be helpful
- Willingness to have BHIP clinicians periodically contacted to complete brief, voluntary, and confidential surveys and semi-structured qualitative interviews related to delivering BHIP-CCM care. Potential participants will be informed that they may decline to participate without any negative repercussions.

**Concurrences:**

____________________________________________

**Name**

VAMC Director	date

_____________________________________________

**Name**

VAMC Chief of Staff	date

_____________________________________________

**Name**

VAMC Mental Health Service Line Lead	date

_____________________________________________

**Name**

VISN Mental Health Lead	date

______________________________________________

**Christopher Miller, PhD**

BHIP-CCM Enhancement Project 2.0 Lead	date
